# Distinct and Diverse: Range-Wide Phylogeography Reveals Ancient Lineages and High Genetic Variation in the Endangered Okapi (*Okapia johnstoni*)

**DOI:** 10.1371/journal.pone.0101081

**Published:** 2014-07-09

**Authors:** David W. G. Stanton, John Hart, Peter Galbusera, Philippe Helsen, Jill Shephard, Noëlle F. Kümpel, Jinliang Wang, John G. Ewen, Michael W. Bruford

**Affiliations:** 1 School of Biosciences, Cardiff University, Cardiff, United Kingdom; 2 Lukuru Foundation, Projet Tshuapa-Lomami-Lualaba (TL2), Kinshasa, Democratic Republic of Congo; 3 Centre for Research and Conservation, Royal Zoological Society of Antwerp, Antwerp, Belgium; 4 Conservation Programmes, Zoological Society of London, London, United Kingdom; 5 Institute of Zoology, Zoological Society of London, London, United Kingdom; Institute of Biochemistry and Biology, Germany

## Abstract

The okapi is an endangered, evolutionarily distinctive even-toed ungulate classified within the giraffidae family that is endemic to the Democratic Republic of Congo. The okapi is currently under major anthropogenic threat, yet to date nothing is known about its genetic structure and evolutionary history, information important for conservation management given the species' current plight. The distribution of the okapi, being confined to the Congo Basin and yet spanning the Congo River, also makes it an important species for testing general biogeographic hypotheses for Congo Basin fauna, a currently understudied area of research. Here we describe the evolutionary history and genetic structure of okapi, in the context of other African ungulates including the giraffe, and use this information to shed light on the biogeographic history of Congo Basin fauna in general. Using nuclear and mitochondrial DNA sequence analysis of mainly non-invasively collected samples, we show that the okapi is both highly genetically distinct and highly genetically diverse, an unusual combination of genetic traits for an endangered species, and feature a complex evolutionary history. Genetic data are consistent with repeated climatic cycles leading to multiple Plio-Pleistocene refugia in isolated forests in the Congo catchment but also imply historic gene flow across the Congo River.

## Introduction

The okapi (*Okapia johnstoni*) is an evolutionarily distinctive even-toed ungulate endemic to the Democratic Republic of Congo (DRC) that has recently been reclassified as ‘Endangered’ by the IUCN [1]. The okapi also holds iconic status among the Congolese people, appearing on bank notes and as the icon of the Congolese conservation agency (the ICCN; Institut Congolais pour la Conservation de la Nature), and is thus a potentially important conservation flagship and umbrella species for the region. However, the species is under major on-going threat from habitat fragmentation, human encroachment, regional armed conflict and poaching [2]. The okapi was recognised as a member of the Giraffidae family in 1901 [3] and to date has only been the subject of one long-term *in situ* ecological study [4]. No photograph of a live, free-ranging, wild okapi was believed to be in existence until the release of a camera-trap image in 2008 [5]. The enigmatic nature of this species is due to its elusive behaviour, affinity for dense rainforest, and the on-going political instability in the regions of the DRC where it occurs, severely limiting scientific study. One important component in conservation management of endangered species is an understanding of the genetic structure of species and populations. This includes an understanding of the causes of any observed genetic differentiation, such as major geographic and demographic barriers in the ancient and recent past [6]. Virtually nothing is known of the diversity or details of the evolutionary history of the *Okapia* genus, which has almost no fossil record, a likely consequence of the okapi's adaptation to closed-canopy forest where the conditions for fossilisation are poor [7]. Although there is a paucity of phylogeographic studies within the Congo Basin, several studies have been carried out on related taxa, across a broader geographic region within Africa [8]–[14]. Including these other realated taxa in a comparative phylogeographic approach can help contextualise the history and diversity of each of the taxa, and help asses the wider implications of the findings.

**Figure 1:**
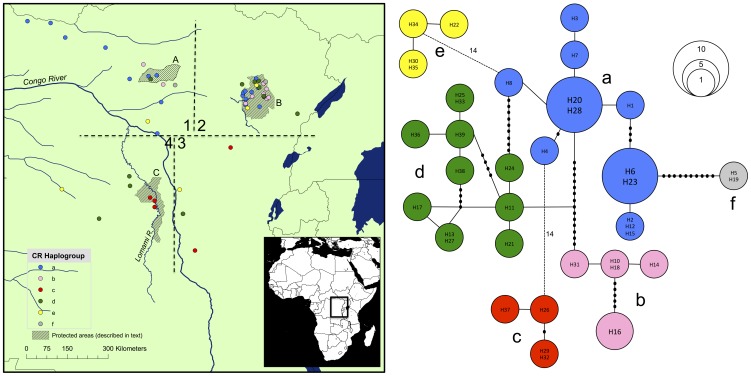
Okapi samples used in the present study, with the colour relating to the adjacent network [30], based on 833 bp of mitochondrial DNA. For the network, TCS connected alleles with a 95% confidence limit, those that did not fall within that limit are connected with dotted lines (with numbers corresponding to the number of mutations). Haplotypes are grouped into haplogroups (number of mutations within always less than between a haplogroup) by colour. Some haplotypes contain more than one label due to different programs using missing data in different ways. Sampling locations are arbitrarily labelled 1–4 for reference in the text. Key protected areas are labelled A (Rubi-Tele Hunting Reserve), B (Okapi Faunal Reserve, RFO), C (Lomami National Park), D (Lomami River).

The historic range of the okapi is thought to have included large sections of the central/eastern Congo Basin, although it is likely that they are currently confined to a small fraction of their former distribution [15]. This relatively wide historic range potentially makes them an important model for investigating historical processes governing the biogeography of the fauna of this region, a subject that remains under considerable debate [16]–[18]. A phylogeographic approach can give insights into gene-flow, divergence times and effective population sizes, which has been done a number of times with widely distributed African species [9], [11], [12], [19]–[21], but comprehensive investigations within the Congo Basin have been much less common [22], [23]. This may be particularly useful in the absence of fossils. The Congo appears to have a profound effect in partitioning faunal diversity. For example, the river is implicated in maintaining one million years of evolutionary divergence between chimpanzees and bonobos [23]–[26], and is thought to be the most important feature for structuring species diversity of *Praomys* spp. (family: Muridae) in the Congo Basin [27]. Many questions regarding central African biogeography cannot, however, be resolved currently due to a paucity of studies. In particular, there are very few studies investigating the role of the Congo River on *within* species genetic diversity [27]. Okapi are a potential model large mammal to help test competing biogeographic theories, and investigate the role of the Congo River on within species genetic diversity due to the okapis close association with closed-canopy rainforest and relatively wide historic distribution (compared to other studied taxa) across the Congo Basin, including both sides of the Congo River (Fig. 1).

Here we used a comparative phylogeographic approach, utilising mitochondrial and nuclear DNA sequences to provide the first molecular-informed description of the evolution of the okapi, and to investigate biogeographic hypotheses in the Congo Basin.

## Methods

### Study area and sampling

This study analysed 69 okapi samples, including feces (n = 37), museum specimens (n = 19 preserved skin samples; sampled with permission from the museum of Central Africa, Tervuren, Belgium; museum sample numbers: 12604, 8305, 14235, 14906, 12517-a, 14454, 11043, 1193, 8011, 9726, 9727, 13991, 13242, 14236, 14234, 909, 13336, 15298, 15299) and clippings of dried skin (n = 13) from artefacts found in villages in the DRC (Fig. 1). The sampling methods used in the present study were therefore all non-invasive. Permission for sampling was provided by the Institut Congolais pour la Conservation de la Nature (ICCN; permit numbers: 0996/ICCN/DG/ADG/MG/KBY/2011 and 090/ICCN/ADG/DG/KV/2012). Fecal samples were collected either by, a) walking randomly placed transects through forest sites and collecting any feces observed, or b) by identifying okapi sign and searching the surrounding area for feces. Sampling methodology a) was used in areas of high okapi density (the Okapi Faunal Reserve [RFO; Fig. 1]), and sampling methodology b) was used in areas of low okapi density (everywhere else in the range that fecal samples were found). Skin samples were in the form of clippings taken from skins owned by individuals living in villages in, or near field sites. Museum samples (skin and bone) were sampled with permission from the Royal Museum of Central Africa, Tervuren. These samples were collected between September 1911 and May 1939 and their locations were obtained from information accompanying the samples, usually the name of a town/village, likely representing the closest habitation to where the individuals were hunted. Samples were grouped into one of four broad sampling ‘regions’ (see Fig. 1) for later analysis and as a descriptive reference.

### Molecular methodology

Five pairs of mitochondrial DNA primers were designed using an available okapi sequence (Genbank accession number: NC_020730.1; OJ1-F [15162–15180]: ATGAATCGGAGGACAACCA, OJ1-R [15359–15380]: GGCCTCTTCTTTGAGTCTTAGG, 217 bp; OJ2-F [15359–15380]: CCTAAGACTCAAAGAAGAGGCC, OJ2-R [15525–15542]: TGCTGCGTTAAGGCTGTG, 184 bp; OJ3-F [15495–15515]: CCCACAACAACCAACACAAAC, OJ3-R [15741–15761]: CGGGATACGCATGTTGACRAG, 247 bp; OJ4-F [15645–15665]: ATATGCCCCATGCATATAAGC, OJ4-R [15885–15905]: CCCTGAAGAAAGAACCAGATG, 263 bp; OJ5-F: CTACCATGAGGACAAATATCATT, OJ5-R: CATTCAGGTTTGATATGAGG). Mitochondrial DNA PCR was carried out with a total volume of 25 µl with 4 µg BSA (New England Biolabs, Ipswich, MA, USA), 1× PCR buffer (Invitrogen, Merelbeke, Belgium), 2.5 mM MgCl_2_, 0.2 mM dNTPs, 0.5 µM each primer, 1 unit of GoTaq (Invitrogen) and 2 µl of DNA. PCR conditions were as follows: 94°C for 3 mins; 60× cycles of 94°C for 30 secs, 58°C for 35 secs, 72°C for 45 secs; and a final extension of 72°C for 5 mins. PCR products were visualised on a 3% agarose gel and sequenced by Macrogen Europe. Of the 69 samples, a subset of 28 were used to produce nuclear intron EPIC [28] sequences. Twelve pairs of nuclear DNA primers were designed (Table S1) from forty-eight primer pairs (selected from the ‘best 96’ loci from Aitken et al. [28]), tested on DNA extracted from blood samples of two captive individuals (White Oaks Conservation Center, Forida; Studbook numbers: 486 and 578; individuals not used for subsequent analysis), preferentially choosing loci that were reported to amplify a single band in *Bos taurus*. PCR conditions followed Aitken et al. [28]. PCR products were visualised on a 3% agarose gel, and primers that produced a single band (n = 20) were tested in four dried skin samples from wild okapi (two from region one; one from region three; and one from region four). PCR mix and conditions were the same as Aitken et al. [28], except annealing times were increased to 1 min and the number of cycles on the second step of the touchdown PCR was increased to 40. PCR products for all four samples were sequenced (Eurofins MWG Operon, Ebersberg, Germany) in the forward and reverse direction. Primers were redesigned to amplify shorter fragments (∼100 bp) for use with non-invasive samples in fragments that contained at least one SNP in the four samples that they were tested in (n = 14). All 14 primer pairs were then tested in 6 non-invasively collected samples (feces and dried skins), and the 12 most consistent primer pairs were selected. These primer pairs were then used for the full set of 28 samples in this study.

### Sequence analysis

Sequences were aligned in Sequencher 4.9 [29] and four mitochondrial DNA contig alignments were created. These consisted of 370 bp of the Cyt *b* gene, the tRNA-Pro (66 bp) and tRNA-Thr (69 bp) genes and 328 bp of the CR, as well as a concatenation of all four genes (833 bp). Contigs consisted of shorter fragments than the original PCR product amplified in order to minimize missing data. To visualise the sequence data, a network of the complete mtDNA fragment was drawn in TCS 1.21 [30]. Pairwise and average nucleotide diversities were calculated in DAMBE v4.2.13 [31], as were amino acid translations for Cyt *b* sequences, and haplotype diversities were calculated in DNAsp v5 [32]. The presence of nuclear inserts of mitochondrial DNA (*Numt* [33] [33]) was assessed by, i) the presence of a single band on an agarose gel, ii) comparison with known mitochondrial DNA sequence on GenBank, iii) for Cyt *b* sequences: the lack of stop codons in the translated amino acid sequence and the lack of any markedly distinct amino acid substitutions. In all cases, SNPs were scored manually in Sequencher 4.9 [29] by sequencing individual PCR amplicons in the forward and reverse direction. To characterise population genetic diversity for this set of 12 EPIC loci, we calculated summary statistics in Arlequin v3.5 [34], using the full set of 28 samples.

Okapi mitochondrial CR nucleotide diversity (π) was compared to CR sequence diversity of a number of other African ungulates. The taxa compared were hartebeest (*Alcelaphus buselaphus spp*; six subspecies), bontebok (*Damaliscus pygargus*), giraffe (*Giraffa camelopardalis spp*; six subspecies), roan antelope (*Hippotragus equinus spp*; five subspecies), African buffalo (*Syncerus caffer*), common eland (*Taurotragus oryx*) and bushbuck (*Tragelaphus scriptus spp*; 21 subspecies), chosen based on the availability of CR sequences in Genbank (studies and GenBank IDs are given in Table S2). The sequences from all taxa were aligned, including any flanking regions, and the start position of the CR for this complete contig was identified, based on the annotation from GenBank. This was to ensure that a homologous section was being compared between taxa. A ubiquitous section of the complete contig was then separated out into the taxon groups shown in Table 1 and re-aligned. This re-alignment consisted of between 268–275 bp for each taxon, including indels. Indels were included in all π calculations. π and SD for Position 31–306 of the CR was calculated for each of the eight taxon groups described in Table 1, in DAMBE v4.2.13 [31]. The contigs for each of these taxon groups are given in Files S1, S2, S3, S4, S5, S6, S7, S8.

**Table 1:** Nucleotide diversity in 268–275 bp of homologous CR sequences of African ungulates, sorted on pi.

Taxon	pi	SD	Number of haplotypes
***Tragelaphus scriptus spp***	**0.151295**	**0.072901**	**197**
*Tragelaphus stricptus barkeri*	0.092222	0.044905	5
***Alcelaphus buselaphus spp.***	**0.085421**	**0.041796**	**92**
*Tragelaphus stricptus roualeyni*	0.075681	0.037075	13
*Tragelaphus stricptus massaicus*	0.069069	0.033945	18
*Alcelaphus buselaphus lichtensteini*	0.058252	0.028857	56
*Tragelaphus stricptus scriptus*	0.056282	0.027889	28
*Tragelaphus stricptus sylvaticus*	0.053813	0.02672	11
***Giraffe camelopardalis spp.***	**0.051564**	**0.025654**	**29**
*Giraffa camelopardalis rothschildi*	0.050059	0.024946	4
***Hippotragus equinus spp.***	**0.046574**	**0.023565**	**43**
*Alcelaphus buselaphus swaynei*	0.04647	0.02328	4
***Syncerus caffer caffer***	0.045189	0.022629	60
***OKAPIA JOHNSTONI***	**0.045015**	**0.022561**	**26**
*Alcelaphus buselaphus lelwel*	0.043154	0.021669	5
*Alcelaphus buselaphus major*	0.041606	0.020936	6
*Giraffa camelopardalis giraffa*	0.041507	0.020894	4
*Tragelaphus stricptus dianae*	0.038925	0.019665	4
*Giraffa camelopardalis reticulata*	0.03833	0.019387	8
***Taurotragus oryx***	**0.037956**	**0.019322**	**50**
*Tragelaphus stricptus knutsoni*	0.037463	0.018972	23
*Tragelaphus stricptus fasciatus*	0.037455	0.018967	5
*Tragelaphus strepsiceros strepsiceros*	0.035514	0.01807	24
*Tragelaphus stricptus heterochrous*	0.034188	0.017418	3
*Tragelaphus stricptus meruensis*	0.033939	0.0173	3
*Alcelaphus buselaphus caama*	0.033708	0.017214	10
*Alcelaphus buselaphus cokei*	0.030588	0.015737	11
*Giraffa camelopardalis tippelskirchi*	0.029038	0.014982	11
*Tragelaphus stricptus sassae*	0.024374	0.012757	8
*Hippotragus equinus cottoni*	0.021818	0.011541	3
*Tragelaphus stricptus pictus*	0.021818	0.011541	3
*Tragelaphus stricptus decula*	0.01705	0.009268	8
*Tragelaphus stricptus meridionalis*	0.014545	0.00807	3
*Tragelaphus stricptus bor*	0.013857	0.00774	5
*Tragelaphus stricptus meneliki*	0.012165	0.006927	9
*Giraffa camelopardalis angolensis*	0.011679	0.006697	5
*Tragelaphus stricptus cottoni*	0.007273	0.004552	7
*Tragelaphus stricptus dama*	0.007273	0.004552	3
*Tragelaphus stricptus johannae*	0.007273	0.004552	5
*Tragelaphus stricptus phaleratus*	0.007273	0.004552	4
***Damaliscus pygargus***	**0.004902**	**0.003371**	**3**
*Giraffa camelopardalis antiquorum*	0.004866	0.003356	3

Key taxa are shown in bold. These often correspond to the combined calculations for several species or subspecies.

### Partitioning of genetic diversity

To partition relative contributions of genetic diversity, AMOVA statistics were calculated using Arlequin v3.5 [34] between the four sampling regions defined in Fig. 1. These regions were designed to compare the extent of genetic structure between individuals at spatial extremes of the okapis range on the same, versus opposite sides of the Congo, in order to investigate the influence of this River on the genetic structure of fauna in the Congo Basin. The sampling regions comprised the Rubi-Tele Hunting Reserve and surrounding areas (region one), the RFO and surrounding areas (region two), the Aruwimi/Lindi/Tshopo (ALT) Rivers and Maiko National Park (region three) and the Tsuapa/Lomami/Lualaba (TL2) Rivers (region four). In order to asses the importance of how these sampling regions were delineated, the AMOVA analyses were repeated, with the line that separates sampling region one and two moved 200 km East, and 200 km West (from the mid-point between the two closest samples between each group). The lines delineating sampling region three could not be moved due to low sample number from this region. The lines delineating sample region four could not be changed as this sampling region is naturally delineated by the Congo River. All 69 mtDNA sequences were used for this AMOVA analysis.

A total of 28 individuals from the four sampling regions (region one, n = 5; region two, n = 14; region three, n = 4; region four, n = 5) were used with the EPIC loci. Of the twelve EPIC loci investigated, four contained greater than one SNP. All SNPs within one sequence were presumed to be linked. Therefore, for analyses using only SNPs (i.e. not the intron sequences), one SNP with high polymorphism was chosen from each of those three intron sequences. AMOVA statistics and F-statistics were calculated on the SNP data using Arlequin v3.5 [34] with the same “groups” and “populations” used for the mitochondrial DNA.

### Population and sequence divergences

To investigate inter- and intra-specific okapi mitochondrial lineage divergences, a time-calibrated phylogeny was created for okapi and giraffe, using 505 bp of homologous mtDNA from GenBank (accession numbers in Table S2 and S3), using BEAST v1.7.5 (Bayesian Evolutionary Analysis by Sampling Trees; [35]), with red deer (*Cervus elaphus*) as an outgroup (due to the phylogenetic proximity, but distinctiveness of Cervidae to Giraffidae; [36]). An HKY +Gamma model was used, selected by jModelTest v2.1.1 [37], [38]. The okapi and giraffe tree was constructed using lognormal relaxed and strict clocks (using a mutation rate of 0.1 s/s/MY, used previously for in the Giraffidae family [10]), and with Yule speciation, coalescent constant, coalescent expansion growth and speciation birth death tree models. The most appropriate model was then selected by comparing the 2*ln Bayes factors for these trees, calculated using TRACER v1.5 with 1000 bootstrap replicates. A value of greater than ten was taken as strong evidence for supporting a model, following Kass and Raftery [39]. The MCMC chain was set at 20,000,000 iterations, with three repeats combined to create the final tree. TRACER v1.5 [40] was used to asses the MCMC output of all BEAST runs. Divergence times (and corresponding standard deviations) between okapi and giraffe, and Giraffidae and Cervidae were taken from Hassanin et al. [36]. Hassanin et al. [36] used an extensive dataset which included complete mitochondrial genome alignments for 210 taxa, utilising six fossil calibration points. To contextualise the results of the Giraffidae phylogeny, a phylogeny was also constructed including okapi, giraffe, duiker and bushbuck jointly, using the same approach as for the Giraffidae phylogeny. Pig (*Sus scrofa*) and collared peccary (*Pecari tajacu*) were selected as outgroups, based on the mtDNA phylogeny of Hassanin et al. [36] giving good support for these species occurring within Cetartiodactyla, but outside Ruminantia. The okapi, giraffe, duiker and bushbuck tree used the 274 bp of Cyt *b* sequence that was overlapping between the present study, and the sequences from Genbank (Table S3), and used the node divergence estimates from Hassanin et al. ([36]; Cetartiodactyla mean 86.8, SD 11.5; Giraffidae mean 15.7, SD 3.3; Pecora mean 28.1, SD 4.5; Bovidae mean 20.0, SD 1.9). The short Cyt *b* sequence for this analysis is therefore due to the limited amount of homologous sequences available for comparison.

PopABC [41] was used to model divergence times between present-day okapi populations, as well as to infer present-day and ancestral effective population sizes, using the EPIC and mitochondrial sequences. For the EPIC loci, haplotypes were reconstructed using Phase v2.1.1 [42]. Pairwise analyses were carried out between sampling regions one versus two, two versus four and one versus four (Fig. 1). The number of iterations used was 1e^6^, with the rejection step set at 1e^−5^. In order to determine prior ranges, preliminary runs were carried out, starting with very wide priors and altering them until all the posterior distributions were distinct from the priors (priors are given in Table S4).

## Results

### Primer design

Following Bonferroni correction, none of the nuclear loci were found to be in linkage disequilibrium or to be out of Hardy-Weinberg Equilibrium (HWE). For HWE testing, sampling regions one to four (Fig. 1) were analysed separately. A summary of nuclear SNP variation is given in Table S1.

### Sequence analysis

A network (TCS) of mtDNA is shown in Fig. 1. Six distinct haplogroups were recovered (number of pairwise differences was always higher between haplogroups than between any two haplotypes within a haplogroup), and while some were geographically restricted, most were not. Haplogroups a, b and f were restricted to the northeast side of the Congo River, and haplogroup d was much more common southwest of the Congo River (42.9% of all samples from this side) than northeast (4.3% of all samples from this side). Haplogroup c was found in 42.9% and 4.3% of all samples from the southwest and northeast sides of the Congo River respectively. Haplogroup e was found in 14.3% and 9.7% of all samples from the southwest and northeast sides of the Congo River respectively. The general pattern therefore was one of widespread haplogroups throughout the okapis range, with some spatial patterning relating to the Congo River (Fig. 1).

Haplotype and nucleotide diversities, and number of polymorphic sites for the mtDNA genes investigated in the present study are given in Table S5. A list of sample ID's, GPS coordinates and haplotype information for the mtDNA dataset is also given in Table S6. CR nucleotide diversity was compared to that of a number of other African species (see Table 1). Based on a common 275 bp of CR sequence, the combined bushbuck (*Tragelaphus scriptus* spp.) dataset showed the highest haplotype diversity (0.151), with bushbuck ecotypes showing highly variable nucleotide diversity estimates (0.007–0.092; Table 1). Nucleotide diversity in okapi (0.045) was slightly lower than the combined giraffe dataset (0.052), and very similar to the African buffalo (0.045), and higher than the eland antelope (0.038).

### Partitioning of genetic diversity

Partitioning of mitochondrial and nuclear DNA sequence variation was investigated across the geographic range, between all sampling regions, and between sampling regions on the northeast side of the Congo River versus the southwest side. For mtDNA, 15.34% of the molecular variation was explained between sampling regions (p<0.001) and 11.41% of the molecular variation was explained by grouping the samples either side of the Congo River (p = 0.257). For the SNPs, 4.82% of the molecular variation was explained between sampling regions (p = 0.041) and 4.07% of the molecular variation was explained by grouping the samples either side of the Congo River (p = 0.249). F_ST_ values were high and significant for all pairwise comparisons between sampling regions one, two and four for mtDNA, except for the comparison between sampling region one and two, which was low, but significant (Table 2). F_ST_ values were non-significant for all pairwise comparisons for the SNP dataset (data not shown).

**Table 2:** Pairwise F_ST_ values for mitochondrial DNA.

	Sampling region 1	Sampling region 2	Sampling region 3	Sampling region 4
Sampling region 1	0			
Sampling region 2	0.072**	0		
Sampling region 3	0.247**	0.186**	0	
Sampling region 4	0.310***	0.192**	0.021^NS^	0

***p<0.001,

**p<0.01,

NS = Not Significant.

To investigate the consequence of the choice of delineation of the sampling regions, we repeated the AMOVA analysis with the line that separates sampling regions one and two moved 200 km East and 200 km West. The results of these changes are given in File S9 and did not notably influence the results.

### Population and sequence divergences

**Figure 2:**
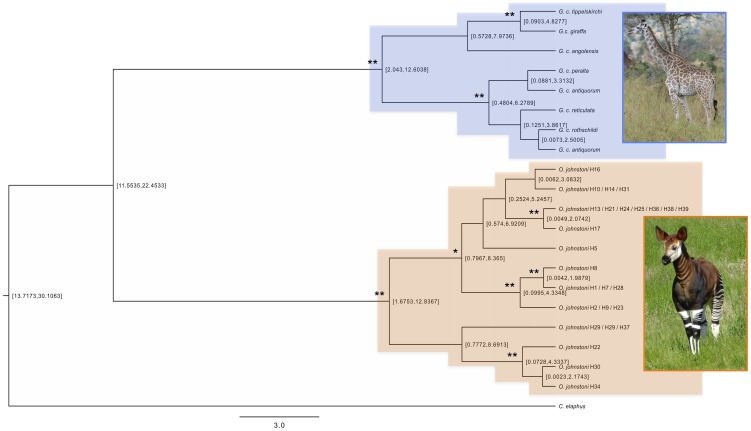
Giraffidae phylogeny drawn in BEAST v1.7.5 [35], with red deer (*Cervus elaphus*) as an outgroup, using 505 bp of mtDNA. Posterior probabilities of >0.8 are highlighted with a single asterisk and posterior probabilities of >0.95 are highlighted with a double-asterisk. Haplotype labels refer to the haplotypes in Fig. 1.

To investigate phylogenetic relationships between haplotypes, BEAST [35] was used to clarify phylogenetic relationships and to infer divergence times of the lineages. In all model comparisons, TRACER identified that a relaxed clock was more appropriate than a strict clock (all 2*ln Bayes factors >10). The Bayes factors for pairwise comparisons of the different relaxed clock models were all low, however in every pairwise comparison, the 2*ln Bayes factor for the Yule speciation model was the highest (min 1.0, max 2.0), and was therefore used. A phylogeny was constructed for okapi and giraffe, using 505 bp of homologous mitochondrial DNA (Fig. 2). The phylogeny identified several deep lineages within okapi, including one ancient divergence that divides okapi mtDNA into two groups. BEAST analysis estimated the most ancestral okapi divergence as occurring at 1.7–12.8 (Fig. 2; 95% HPD; mean, 6.83; posterior probability of 0.96) mya. Six of the ten other okapi divergence events were also estimated at greater than one million years old. The giraffe section of the phylogeny (Fig. 2) showed divergence events of a similar magnitude, with the most ancestral divergence estimated at 2.0–12.6 (95% HPD; mean, 6.3; posterior probability of 0.99) mya.

**Figure 3:**
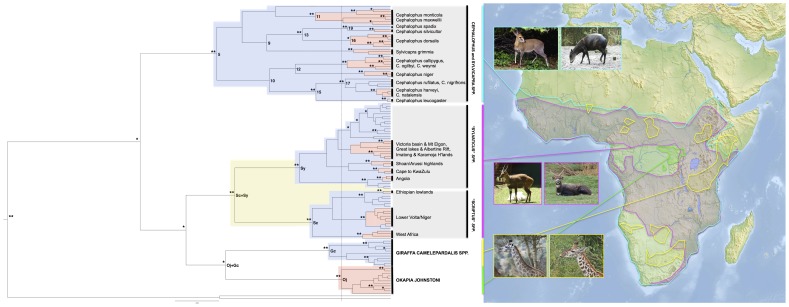
Okapi (*Okapia johnstoni*), giraffe (*Giraffa camelopardalis*), bushbuck (*Tragelaphus scriptus spp.*) and duiker (Cephalophinae *spp.*) tree drawn in BEAST v1.7.5. Posterior probabilities of >0.8 are highlighted with a single asterisk and posterior probabilities of >0.95 are highlighted with a double-asterisk. Dotted line indicates the most ancestral divergence within okapi. The shading on the tree shows when taxonomic units can be monophyletically grouped, with the different colours corresponding to different levels of inclusiveness for these groupings. For example, for bushbuck, Victoria Basin & Mt Elgon, Great Lakes & Albertine Rift and Imatong & Karamoja Highlands ecoregions could be grouped monophyletically, and are shaded red. The next monophyletic taxonomic grouping are the “scriptus” species (shaded blue), and then all bushbuck (shaded yellow).

In order to further understand the okapi phylogeny, trees for okapi, giraffe, duiker and bushbuck, were also reconstructed jointly (Fig. 3). This was done in an attempt to address some of the discrepancies that can be encountered when using dated phylogenies, such as faulty calibration points [43], rate heterogeneity among lineages [44], and time dependent of rates of evolution [45], [46]. The comparative approach addresses these issues by simply providing relative divergence estimates using a single methodology, rather than trying to estimate absolute dates using different methodologies. Despite using only 274 bp of sequence data, the alignment for this phylogeny included 136 variable sites. The phylogeny also contained a large number of nodes supported with a posterior probability of greater than 0.95 (Figure 3). This phylogeny (relaxed lognormal) gave estimates of TMRCA (Time to Most Recent Common Ancestor) for okapi of 2.0–7.9 mya and for giraffe of 2.7–9.3 mya. The topology of the section of the tree containing bushbuck and duiker species was broadly concordant with phylogenies of these species created in previous studies (Moodley and Bruford [11]; Johnston and Anthony [47] respectively). The 95% confidence intervals of the divergence times of the duiker species in the phylogeny from the present study all overlap with the intervals in Johnston and Anthony [47]. However, the inferred dates of the coalescent events of the Cyt *b* lineages for bushbuck, and the *T. scriptus* and *T. sylvaticus* lineages in this study were considerably higher than Moodley and Bruford [11] (Table 3). This difference is likely due to a combination of, 1) the larger mtDNA fragment investigated in that study, 2) the more comparative approach in the present study, utilising a more inclusive taxon set for the phylogeny, 3) the use of different programs for constructing phylogenies between the present study and that one. Based on this joint phylogeny, the divergence of the two most divergent okapi lineages predates the divergence of several major duiker species, including *C. jentinki* from *C. dorsalis* (Fig. 3, node 16); *C. rufilatus* (node 17); *C. nigrifrons* from *C. harveyi* (node 17); *C. natalensis* (node 17); and *C. spadix* from *C. silvicultor* (node 19). These duiker lineages (nodes 16, 17 and 19) have previously been estimated to have diverged between 1.74–3.54, 1.18–2.38 to 0.80–1.91 mya respectively [47]. The divergence of the okapi lineages also appears to pre-date the emergence of many of today's described bushbuck subspecies, for example *T. sylvaticus sylvaticus* from *T. sylvaticus meneliki* and *T. sylvaticus powelli*, and approximately twice as old as the emergence of both *T. scriptus decula* and *T. sylvaticus ornatus*. The TMRCA for the okapi is similar to that of all the giraffe subspecies.

**Table 3:** 95% HPD intervals for dates of divergences (mya) for Fig. 3 of the present study, and from the original studies (nodes 5–17, Johnston and Anthony [48]; nodes Sc/Sy, Moodley and Bruford [14]).

Node	Dates (Previous study; mya)	Dates (present study; mya)
5	6.27–11.43	10.47–20.91
9	4.16–7.78	6.60–16.44
10	3.58–6.69	6.50–16.20
11	2.68–5.31	3.71–10.93
12	2.52–4.97	4.53–13.67
13	2.53–4.93	4.38–12.52
15	2.13–4.27	3.16–11.75
16	1.74–3.54	1.51–6.62
17	1.18–2.38	1.87–7.30
19	0.80–1.91	1.35–7.28
Sc	2.0–3.0	4.01–10.97
Sy	2.0–3.0	4.65–12.65
Sc+Sy	3.9–6.5	9.46–19.01

PopABC [41] was used to infer divergence times, migration rates, and present-day and historic effective population sizes of pairwise combinations of samples from sampling regions one, two and four, using both mitochondrial DNA and nuclear loci (Table 4; for posterior distributions see Files. S10, S11, S12 [A–G]; samples from sampling region three were excluded due to low sample number). Migration rate was inferred to be consistently lower when comparing populations northeast verses southwest of the Congo River compared to the same side, and three of the four inferred migration rates across the Congo were an order of magnitude lower than the two migration rates on the same side. In every instance ancestral effective population size (NeA1) was considerably higher than any of the inferred present-day effective population sizes (Ne1 and Ne2), implying a reduction in population size since these populations became separated. Time since divergence of all the populations was inferred at approximately 200 kya, and interestingly, was the same for all population comparisons. Inferred mutation rates, however, varied substantially among pairwise comparisons, as did the effective population size for region two.

**Table 4:** Values with the highest posterior probabilities for the parameters investigated for the popABC analysis, with comparisons between regions one, two and four (R1, R2 and R4).

Parameter	R1vR4	R1vR2	R2vR4
AvMutS	5e^−4^	<1e^−5^	1.5e^−2^
mig1	0.2	4	1
mig2	0.2	2	0.1
NeA1	6000	4500	14000
t1	2e^5^	2e^5^	2e^5^

Parameters investigated are average mutation of the sequence (AvMutS), migration into each population (mig1 and mig2), effective population size of the ancestral population (NeA1) and time of population splitting (t1). Effective population size of each population (Ne1 and Ne2) were omitted from the table due to lack of convergence.

## Discussion

### Okapi genetic diversity and evolutionary history

Paleontological records of *Okapia spp.* are virtually non-existent, with no known fossils predating the Pleistocene, except *Okapia stillei* (Dietrich [48] in Van der Made and Morales [49]), which has since been reclassified as Giraffa [7]. Giraffidae are first known from the late early Miocene in Africa, and by the Late Miocene giraffids were very widespread and diverse. During the Early Pliocene they became rare in Eurasia, but remained diverse in Africa [7], [50]–[52]. Okapi and giraffe are thought to share a common ancestor approximately 16 mya [36], [53]. Based on the okapi and giraffe phylogeny, the present study estimates the most ancient divergence within okapi mitochondrial lineages to be minimally 1.7 mya (divergence of haplotypes H22, H29, H30, H34 and H37 from the remaining haplotypes [Fig. 2]; with maximum sequence divergences of 7.10% and 3.49% for CR and Cyt *b* sequences respectively). This result implies that okapi mitochondrial DNA haplotype divergence dates to at least the early Pleistocene. Sequence divergences of this magnitude are more consistent with divergence dates detected between African species or subspecies (e.g. divergence of *Elephas* and *Loxodonta* elephant genera [54]; the *Phacochoerus africanus massaicus*, *P. a. sundevallii and P. a. africanus* warthog subspecies divergences [21]; spotted hyena divergences [55]; and the Scriptus and Sylvaticus bushbuck species divergences [11]), yet there is no suggestion that okapis comprise more than one taxon. This estimate of intra-specific divergence time for okapi is also at the upper limit for what has previously been estimated for the emergence of the extant giraffe subspecies (0.54–1.62 mya [10]).

The present study constructed a phylogeny (Fig. 3), and calculated genetic diversities (Table 1) in a comparative manner, that included multiple ungulate taxa. Based on the combined phylogeny, the divergence of the two most ancestral okapi mitochondrial lineages (divergence that splits haplotypes H22, H29, H30, H34 and H37 from the remaining haplotypes) predates the divergence of several major duiker lineages, which have previously been estimated to have diverged between 0.80–3.54 mya. This gives further support to a divergence of at least 1.7 mya for the most ancestral okapi mtDNA lineage. The divergence of okapi is again estimated to be similar to that of all the giraffe subspecies, as well as the emergence of many of the bushbuck subspecies (e.g. *T. scriptus decula* and *T. s. ornatus*). This is a surprising result, particularly when one considers the morphological and geographic variation that is contained within these giraffe, duiker and bushbuck taxa [10], [11], [47]. The results of the CR nucleotide diversity comparison showed similar results. Okapi nucleotide diversity was similar to the combined giraffe subspecies and African Cape buffalo, and higher than the eland antelope. This comparative methodology provides a much more useful and meaningful means of comparing interspecific genetic diversity than simply stating genetic diversities out of context. Table 1 shows okapi to be one of the more genetically diverse of the ungulate species investigated in this study, implying a rich and diverse evolutionary history.

### Evolutionary biogeography of the Congo Basin

The most ancestral mitochondrial DNA divergence in okapi is dated at greater than 1.7 mya (Fig. 2). The Congo River is a likely candidate for the cause of the split of the most ancestral mtDNA sequence lineages in okapi, however, it is not possible to prove this definitively due to the possibility of retention of ancestral polymorphism in populations either side of the Congo River. The mitochondrial DNA network shows six distinct lineages (Fig. 1), and divergences of several of the other major okapi mtDNA lineages from the BEAST phylogeny are also dated at greater than one million years ago (the divergence of both of the monophyletic clades is *at least* 0.8 mya, Fig. 2). These dates may be explained by the Congo Basin fragmenting into refugia at various stages throughout the Pleistocene. This is consistent with a hypothesis of increases in African climate variability [56] and aridity [57] at approximately 2.8–2.5, 1.9–1.7 and 1.1–0.9 mya. Okapi are however known to be highly selective folivores and currently occupy a disjunct distribution within the Congo Basin. Refugia may therefore have provided isolated regions of suitable forest type, rather than simply comprising patches of forest separated by savannah. Cowling et al. [16] simulated the paleovegetation of Central Africa and LGM simulations indicate that although tropical broadleaf forest may not have been severely displaced by expanding grassland in central Africa, the structure of the forests may have been very different from today (with forests characterized by lower leaf area indices, lower tree heights and lower carbon content).

The inferred Approximate Bayesian relative divergence times and migration rates between the okapi sampling regions were relatively consistent. As would be expected, migration rates of sampling regions one versus two (same side of river) were consistently higher than regions one versus four and two versus four (opposite sides of river). Interestingly however, divergence times between all population comparisons were the same (∼200 kya). When taken together, these results imply that although populations on the same side of the Congo River maintained much higher gene-flow since the time of divergence, they nonetheless diverged around the same time to those on opposite sides of the river. This would suggest that initial population divergence between okapi populations either side of the Congo River was primarily linked to the same biogeographic process that separated those populations on the same side of the river. A possible explanation for this could again be forest fragmentation, linked to repeated glaciation events during the Pleistocene [57]–[59]. This estimate of population divergence is considerably more recent than the estimates of sequence divergence discussed earlier (>1.7 mya). This implies greater than one population fragmentation event, again suggestive of repeated cooling events. These results add to a growing body of evidence that tropical forest refugia can play an important role in driving evolutionary diversification, but that this role has been much more prominent in tropical Africa [47], [60]–[64], than in the Amazon [65]–[69].

These results are also in accordance with the distribution of mtDNA haplotypes. Deep genetic divergences indicate historic population isolation, but the presence of these haplotypes on each side of the Congo River suggests relatively recent gene-flow. Bonobos and chimpanzees provide a particularly interesting comparison to okapi, as their combined range spans the Congo River. Bonobos and chimpanzees are estimated to have diverged ∼1 mya [23]–[26], with chimpanzees restricted to the northeast side of the Congo River, whereas bonobos are restricted to the southwest side. The diversification of chimpanzee sub-species, and bonobo haplogroups are explained by fluctuations in climate during the Pleistocene and the associated changes in forest cover [23], [70]. Taken together, these studies suggest periods of Pleistocene forest expansion that genetically differentiated southern, eastern and western populations in large numbers of savannah taxa. This would imply that okapi have at some point in the past been able to either cross or go around the Congo River, allowing admixture between mitochondrial lineages, whereas chimpanzees and bonobos have not. It is currently unknown if okapi are able to cross large rivers, and in the case of the Congo it may intuitively sound unlikely due to the River's considerable size, and as it is likely to have existed roughly in the same formation for tens or even hundreds of millions of years [71]. However, geomorphic mechanisms do exist that may make this possible. Neck cutoff and oxbow lake formation could theoretically allow populations of organisms to move to the opposite side of a river without actually crossing it. A second possible explanation, anastomosis, is a common mechanism where the path of a river is broken into islands with channels of much smaller size. This process could have led to each of the individual channels being surmountable when the entire width of the river is not.

### Partitioning of present-day genetic diversity

We show that deeply divergent mitochondrial haplotypes are ubiquitous across the okapi's range. This suggests that historic biogeographic processes have shaped the structure of genetic diversity in this species, and these processes pre-date the present-day distribution of okapi. Nonetheless, present-day geography also contributes to the structuring of genetic diversity in okapi. AMOVA consistently showed a very high percentage of genetic differentiation between sampling regions. This, in combination with high F_ST_ values for mitochondrial DNA sequence data between populations on the same, and opposite sides of the Congo River, particularly between sampling regions one and four, highlights the importance of the Congo River in structuring present-day genetic diversity in okapi. In comparison, mtDNA F_ST_ values between sampling regions one and two were much lower. The level of present-day population genetic differentiation seen in okapi is within the range of what is seen among chimpanzee populations [70]. No known morphological or behavioral differences separate these chimpanzee populations, however they are regarded as separate sub-species [72], [73], again emphasizing the remarkable genetic diversity seen within okapi.

The findings presented here therefore add to the evidence that a combination of the Congo River [23]–[26], and Pleistocene forest refugia [47], [60]–[64] are the most important factors structuring contemporary genetic diversity of large mammals in the Congo Basin. However, interestingly, the Lomami River (a tributary running parallel southwards with the upper stretches of the Congo River) is the feature that delineates the range of the okapi population on the southwest side of the Congo River. This river has recently been shown to limit the range of a recently described primate, the “lesula” (*Circopithecus lomamiensis*, [74]) and has also recently been shown to be the only river to be a strong barrier to gene-flow in bonobos [23]. A future avenue for research could therefore involve a multi-taxon analysis of the combined role of the Congo and Lomami Rivers in structuring species and genetic diversity in this area.

The future viability of okapi is under considerable doubt [1]. The present study identifies a rich and diverse evolutionary history for this emblematic and elusive species. This is likely a result of a dynamic historical biogeography in the Congo Basin, leading to expansion and contraction of Pleistocene refugia. Contemporary okapi populations contain high levels of genetic diversity, with mtDNA haplotypes widespread. The pinpointing of evolutionarily significant units on the basis of molecular data alone is therefore complex for this species and perhaps not justifiable given the equivocal nature of the data and the overall threats to the species across its range. It is certainly noteworthy, however, that the okapis remaining southwest of the Congo River have divergent allele frequencies and are at low density and we therefore suggest that they be treated as a separate management unit (*sensu* Moritz [75], for example) to the population north of the river, re-emphasising the biogeographic importance of this region (referred to as “TL2” [74]). However, in general conservation efforts should aim to protect as large a proportion of the okapis range as possible.

## Supporting Information

Table S1 **EPIC primers designed and tested in this study.** Observed and expected heterozygosities were generated using the same 28 individuals as the AMOVA and F-statistic analysis in the present study. Multiple SNPs occurring on a single sequence have been notated with a suffixed letter.
(XLSX)

Table S2 **Study names and genbank IDs of sequences used in the comparative analysis of CR nucleotide diversity.** CR section refers to the DNA fragment available for use from genbank, with numbers referring to the position of the fragment, relative to the start of the CR (0) based on the genbank annotations.
(XLSX)

Table S3 **Study names and genbank IDs of sequences used for the 274 bp phylogeny of bushbuck, duiker, giraffe and okapi, including pig and collared peccary outgroups.** Original* and final* haplotypes refer to number of haplotypes in the study in which those haplotypes were originally sequenced, and haplotypes based on 274 bp sequences in the present study respectively.
(XLSX)

Table S4 **Table of prior values for PopABC analyses.** All priors (Ne, effective population size; NeA, ancestral effective population size; t, divergence time; mig, migration rate) used a uniform distribution, except mutAvS (average sequence mutation rate), which PopABC only gives the option of a normal or lognormal distribution. Priors were determined by carrying out preliminary runs, and altering the prior value until all posterior distributions were distinct from the prior distributions.
(XLSX)

Table S5 **Nucleotide and haplotype diversities, and number of polymorphic sites for the mtDNA genes used in the present study.**
(XLSX)

Table S6 **Table containing individual sample ID's, sequence data for the complete 833 bp fragment used in the present study, and GenBank accession numbers for the corresponding submissions to GenBank (submitted separately for the CR haplotypes, and a fragment that contains the Cyt b, tRNA-Thr and tRNA-Pro genes).**
(XLSX)

File S1 **Contig of position 31–306 of the 92 CR haplotypes for** ***Alcelaphus buselaphus*** **.** Nucleotide diversity for these haplotypes is described in Table 1.
(TXT)

File S2 **Contig of position 31–306 of the three CR haplotypes for** ***Damaliscus pygargus*** **.** Nucleotide diversity for these haplotypes is described in Table 1.
(TXT)

File S3 **Contig of position 31–306 of the 29 CR haplotypes for** ***Giraffe camelopardalis*** **.** Nucleotide diversity for these haplotypes is described in Table 1.
(TXT)

File S4 **Contig of position 31–306 of the 43 CR haplotypes for** ***Hippotragus equinus*** **.** Nucleotide diversity for these haplotypes is described in Table 1.
(TXT)

File S5 **Contig of position 31–306 of the 26 CR haplotypes for** ***Okapia johnstoni*** **.** Nucleotide diversity for these haplotypes is described in Table 1.
(TXT)

File S6 **Contig of position 31–306 of the 60 CR haplotypes for** ***Syncerus caffer*** **.** Nucleotide diversity for these haplotypes is described in Table 1.
(TXT)

File S7 **Contig of position 31–306 of the 50 CR haplotypes for** ***Taurotragus oryx*** **.** Nucleotide diversity for these haplotypes is described in Table 1.
(TXT)

File S8 **Contig of position 31–306 of the 197 CR haplotypes for** ***Tragelaphus scriptus*** **.** Nucleotide diversity for these haplotypes is described in Table 1.
(TXT)

File S9 **Description of AMOVA analysis, repeated after sampling region delineations had been changed.**
(DOCX)

File S10 **Prior (black) and posterior (blue) distribution for PopABC** [41] **analysis of region one versus region two (** **Fig. 1** **).** Parameters investigated are mutation rate (mut rate [A]), migration into sampling regions one and two (labelled mig1 [B] and mig2 [C] respectively), effective population size of sampling regions one and two (labelled Ne1 [D] and Ne2 [E] respectively), effective population size of the ancestral population (NeA [F]) and time since divergence of sampling regions one and two (labelled t1 [G]).
(DOCX)

File S11 **Prior (black) and posterior (blue) distribution for PopABC analysis of sampling region one versus sampling region four.** Parameters investigated are mutation rate (mut rate [A]), migration into sampling regions one and four (labelled mig1 [B] and mig2 [C] respectively), effective population size of sampling regions one and four (labelled Ne1 [D] and Ne2 [E] respectively), effective population size of the ancestral population (labelled NeA [F]) and time since divergence of sampling regions one and four (labelled t1 [G]).
(DOCX)

File S12 **Prior (black) and posterior (blue) distribution for PopABC analysis of region two versus region four.** Parameters investigated are mutation rate (mut rate [A]), migration into sampling regions two and four (labelled mig1 [B] and mig2 [C] respectively), effective population size of sampling regions two and four (labelled Ne1 [D] and Ne2 [E] respectively), effective population size of the ancestral population (labelled NeA [F]) and time since divergence of sampling regions two and four (labelled t1 [G]).
(DOCX)
