# Supplementary material for: Distinct and Diverse: Range-Wide Phylogeography Reveals Ancient Lineages and High Genetic Variation in the Endangered Okapi (Okapia johnstoni)
Source: PLoS One. 2014 Jul 9;9(7):e101081. doi: 10.1371/journal.pone.0101081 (PMC4090074; doi:10.1371/journal.pone.0101081)
Supplement: File S9 — Description of AMOVA analysis, repeated after sampling region delineations had been changed. (DOCX) [file pone.0101081.s015.docx]

**File S9** Description of AMOVA analysis, repeated after sampling region delineations had been changed

In order to asses the importance of how the sampling regions (sampling regions 1-4, Figure 1) in the present study were delineated, the AMOVA analyses were repeated, with the line that separates sampling region one and two moved 200 km East, and 200 km West (from the mid-point between the two closest samples between each group). For the mtDNA dataset, the maximum change is percentage of variation explained between sampling regions was only +3.46% and the level of significance (p < 0.001) did not change, when the sampling region delineation was moved. The maximum change is percentage of variation explained by grouping samples either side of the Congo River was only -3.13%, when the sampling region delineation was moved. For the SNP dataset, the maximum change is percentage of variation explained between sampling regions was only +0.75% (p-value still < 0.05). The maximum change is percentage of variation explained by grouping samples either side of the Congo River was only -0.63% (still not significant). In terms of changes in F_ST_ values when sampling region delineations were changed, for mtDNA the lowest F_ST_ value was still between sampling region one and two (F_ST_ = 0.068 and 0.146) and the highest was still between sampling region one and four (F_ST_ = 0.279 and 0.300). All F_ST_ values that were previously significant remained significant and all that were not significant remained so. For the SNP dataset, all FST values remained non-significant, except between sampling regions one and three and one and four, which became marginally significant (p < 0.05). These minor changes do not therefore alter any of the conclusions in the present study.
