# Supplementary material for: Distinct and Diverse: Range-Wide Phylogeography Reveals Ancient Lineages and High Genetic Variation in the Endangered Okapi (Okapia johnstoni)
Source: PLoS One. 2014 Jul 9;9(7):e101081. doi: 10.1371/journal.pone.0101081 (PMC4090074; doi:10.1371/journal.pone.0101081)
Supplement: File S11 — Prior (black) and posterior (blue) distribution for PopABC analysis of sampling region one versus sampling region four. Parameters investigated are mutation rate (mut rate [A]), migration into sampling regions one and four (labelled mig1 [B] and mig2 [C] respectively), effective population size of sampling regions one and four (labelled Ne1 [D] and Ne2 [E] respectively), effective population size of the ancestral population (labelled NeA [F]) and time since divergence of sampling regions one and four (labelled t1 [G]). (DOCX) [file pone.0101081.s017.docx]

**File S11 [A-G].** Prior (black) and posterior (blue) distribution for PopABC analysis of sampling region one versus sampling region four. Parameters investigated are mutation rate (mut rate [A]), migration into sampling regions one and four (labelled mig1 [B] and mig2 [C] respectively), effective population size of sampling regions one and four (labelled Ne1 [D] and Ne2 [E] respectively), effective population size of the ancestral population (labelled NeA [F]) and time since divergence of sampling regions one and four (labelled t1 [G]).

G

F

E

D

C

B

A

References

1. Lopes JS, Balding D, Beaumont M (2009) PopABC: a program to infer historical demographic parameters. Bioinformatics 25: 2747-2749.
